# Sexual and Urinary Dysfunction Following Isolated Acetabulum Fractures: A Systematic Review of the Literature

**DOI:** 10.3390/jcm14010230

**Published:** 2025-01-03

**Authors:** Sophia M. Wakefield, Nikolaos K. Kanakaris, Peter V. Giannoudis

**Affiliations:** 1 Academic Department of Trauma & Orthopaedics, School of Medicine, University of Leeds, Leeds LS2 9LU, UK; sophwakey@gmail.com (S.M.W.); n.kanakaris@nhs.net (N.K.K.); 2 NIHR Leeds Biomedical Research Centre, Chapel Allerton Hospital, Leeds LS2 9LU, UK

**Keywords:** acetabulum, fracture, sexual function, urinary function

## Abstract

**Background/Objectives:** Acetabular fractures are rare fractures of the pelvis which usually result from trauma. Whilst data are reported on sexual and genitourinary function in those with pelvic fractures, less is known about those with isolated acetabulum fractures. This systematic review aimed to determine, first, the frequency of sexual and genitourinary dysfunction following isolated acetabulum fractures and, second, the nature of these complications. **Methods:** A PRISMA strategy was used. Medline, Cochrane Central Register of Controlled Trials, Scopus, and EMBASE library databases were interrogated using pre-defined MeSH terms and Boolean operators. Quality of evidence was evaluated based on OCEBM and GRADE systems. **Results:** Seven studies based on 648 individuals were identified with a mean follow-up time of 33.6 ± 22.4 months. Five papers described sexual functional outcomes, and two reported genitourinary function. Acetabulum fractures were noted to have an impact on sexual function ranging from 20.0% to 39.8% within the groups analysed. With respect to genitourinary outcomes, incidence of lower urinary tract injury and spontaneous voiding failure was quite low, but due to the existence of little data, firm conclusions cannot be made. **Conclusions:** This review has highlighted a paucity of data related to outcomes of sexual and genitourinary function in patients who are post-acetabulum fracture. The limited available data suggests that acetabular fractures have an impact on sexual function, but the impact on genitourinary function is less clear. Further prospective work is required to better understand the relationship between baseline demographics, injury characteristics, injury mechanism and concurrent injuries, and surgical fixation and acetabular-fracture outcomes.

## 1. Introduction

Acetabulum fractures are a type of pelvic fracture which may occur as isolated fractures or in combination with other pelvic fractures. Overall, they are a relatively uncommon type of fracture with reported incidences ranging from 3 to 9.5 out of 100,000 [1,2,3,4]. Younger patients tend to be male, with healthy bone structure, and have injuries sustained after a high-energy impact, either after a road traffic accident (RTA), a fall from a height of more than two metres (>2 m) or following contact sport [5,6]. In contrast, older patients are more likely to be female, have pre-existing bone fragility, and sustain their injuries after a low-impact fall (less than 1 m) [7].

Immediate fracture management requires adequate pain relief, fracture reduction, good bone alignment and fixation, in addition to early rehabilitation to prevent long-term progression to secondary osteoarthritis (OA) and total hip arthroplasty (THA) [8,9,10]. The decision to operate is largely dependent on the preferences of the surgeon and the patient, being influenced by a number of factors including fracture type and severity, associated injuries, co-morbidities and pre-injury functional status [11]. A number of different surgical fixation approaches are employed, the most common of which are the ilioinguinal (IL), modified Stoppa and Kocher–Langenbeck (KL) [12]. A number of general complications occur following acetabular fractures which include blood loss, surgical site infection, deep vein thrombosis, muscle weakness, heterotopic ossification, and post-traumatic OA of the hip [13,14]. However, additional problems may arise from the surgical technique itself [15].

Interestingly, to date, most published studies have focussed on pelvic fractures as a whole rather than specifically on the acetabulum. Metze et al. (2007) reported a range of male sexual function-related symptoms including ejaculatory dysfunction, loss of sensation, painful erection, and a restricted range of sexual positions [16]. The authors also noted that more severe pelvic fracture patterns were associated with more severe complications. Vallier et al. (2012) highlighted an association between dyspareunia, functional outcome, and quality of life (QoL) [17]. Harvey-Kelly et al. (2014) reported sexual dysfunction in both sexes in 12–72% of patients [18] with 38–56% experiencing dyspareunia [18].

The genitourinary system is also at potential risk following pelvic and acetabulum fractures due to their relatively close proximity to the bladder and urethra [19]. These soft tissue structures may be affected at the time of injury through direct impact, abrasion against sharp bony fragments, or due to disruption of the nerve or vascular supply to the organs themselves [15,20]. Injury may also be a consequence of the surgery itself. Esparaz et al. (2015) reported that the pelvis and acetabulum are one of the most common causes of iatrogenic genitourinary injury [21]. Testa et al. (2022) noted that whilst urologists are very familiar with navigating through pelvic soft tissues, this is not necessarily so for orthopaedic surgeons [22]. The most commonly described urogenital injury following pelvic fractures relates to the bladder [23]. Different surgical approaches may also place the patient at risk; for example, when the modified Stoppa approach is performed, the space between the pubic symphysis and anterior bladder (known as the ‘space of Retzius’) is explored, making it vulnerable to injury [24,25]. As a result, surgeons may use strategies, such as a sponge or a balloon catheter to protect the bladder [26]. In general, iatrogenic damage to the urethra and ureters appears relatively rare [27].

This systematic review sought to determine the degree of sexual and genitourinary function in patients following an acetabulum-specific fracture. The primary objectives were to evaluate the frequency of sexual and genitourinary dysfunction following isolated acetabulum fractures and, second, to determine the nature of these complications.

## 2. Materials and Methods

### 2.1. Search Strategy and Criteria

This systematic review was structured using the Preferred Reporting Items for Systematic reviews and Meta-Analysis (PRISMA) guidelines [28] (Table S1). The protocol of this review was established prior to the extraction of data. This review was not registered on a systematic review database. A list of Medical Subject Heading (MeSH) terms and Boolean operators were collected: (acetabulum OR acetabular) AND (fracture) AND (sexual function* OR erectile function* OR urinary function*). These terms were used to search Medline (through PubMed), Cochrane Central Register of Controlled Trials (CENTRAL), Scopus, and EMBASE databases. The search strategies utilised are described in Table S2 and were performed in September 2024.

### 2.2. Selection of Studies

A Population Intervention Comparison Outcome (PICO) approach [29] was used to define the inclusion criteria. *Population*: Adults (over 18 years old) with acetabulum fractures. Limits were not imposed on biological sex, ethnicity or co-morbidities of the included individuals. *Intervention*: surgical fixation of acetabular fractures. *Comparator*: non-operative management of acetabular fractures. *Outcomes*: the primary outcomes measured were genitourinary and sexual function. Exclusion criteria included: reviews, editorials and conference abstracts, subjects aged 16 years and below, studies evaluating pelvic fractures not restricted to the acetabulum, studies evaluating outcomes which do not describe genitourinary function, and cadaveric studies.

No restrictions were placed on language or date of publication, and, therefore, all studies were considered for eligibility. Prior to full inspection, both titles and abstracts of studies were screened. All eligible studies were evaluated and reference lists reviewed; this was to ensure no articles were overlooked from the initial database search. The full texts of all studies meeting the inclusion criteria of this review were obtained following the removal of duplicate articles. Two reviewers blindly performed the study selection and extraction of data to increase the reliability of data collection. Discussion with a senior author resolved any disagreements between reviewers.

### 2.3. Collection of Data

Following the extraction of data, a purpose-designed Microsoft Excel Spreadsheet was used to synthesise findings. Collated data included the following: (1) study characteristics (study design, sample size); (2) patient demographics and baseline characteristics (age, biological sex, ASA grade); (3) initial acetabulum injury prior to surgical intervention (mechanism of injury, injury severity score (ISS), Judet–Letournel classification, fracture pattern); (4) surgical procedure performed; (5) outcome measures (radiographic outcomes, bony union, functional outcomes, sexual function, urinary function, complications); and (6) follow-up time.

### 2.4. Methodological Quality Assessment

OCEBM ‘Levels of Evidence’ guidelines [30] were utilised to assess and grade the methodological quality of the studies included within this systematic review. In addition, the Grading of Recommendations, Assessment, Development, and Evaluation (GRADE) system [31] was used to evaluate the overall quality of evidence. Recommendations were graded as either High, Moderate, Low, or Very Low; this was based on the authors’ interpretation of the true effect versus the estimated effect of the included studies. Criteria for risk of bias, imprecision, inconsistency, indirectness, and publication bias were used to grade the evidence.

### 2.5. Statistical Analysis

This study utilised descriptive statistical analysis (e.g., mean ± standard deviation (SD), ranges, percentages, 95% confidence intervals (CIs), and ratios).

## 3. Results

### 3.1. Search Results

**Figure 1:**
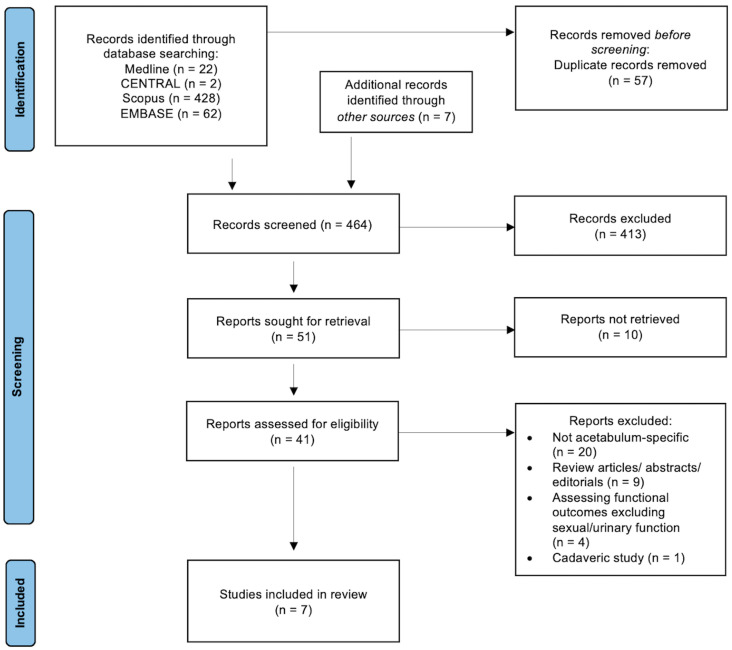
Preferred reporting items for systematic reviews and meta-analyses (PRISMA) flowchart.

The PRISMA flowchart is shown in Figure 1.

A total of 22 Medline articles, 2 CENTRAL articles, 428 Scopus articles, and 62 EMBASE articles were retrieved. An additional search generated a further 7 studies, providing a total of 521 baseline articles. Once the removal of duplicate articles between databases was complete, 464 articles were screened. Following title and abstract screening, these studies were further narrowed to 51, with an overall 41 articles assessed for study eligibility. In total, 7 studies met the inclusion criteria and were therefore obtained for review [32,33,34,35,36,37,38].

An overview of the studies included in the systematic review is provided in Table 1. All seven studies included in the analysis were primary research articles and included 648 individuals treated following an isolated acetabular fracture [32,33,34,35,36,37,38].

### 3.2. Methodological Quality

OCEBM ‘Levels of Evidence’ (Table S3) highlighted the level of evidence of all seven studies being either “Level II” [36,37,38], or “Level III” [32,33,34,35]. This is due to all research being either retrospective or prospective cohort studies.

In addition, Table S3 provides an overview of the GRADE analysis assessment, which demonstrated the quality of evidence to be “Low” for all analyses [32,33,34,35,36,37,38], as there were no randomised controlled trials or large observational studies included, and the data reported was incomplete, thus, making it difficult to estimate the true effect.

### 3.3. Patient Demographics and Baseline Characteristics

Of the 648 individuals reported in this review, 203 were men and 56 were women; the sexes of 389 cases were unreported (Table 1). Not all studies presented mean age, but the broad mean age range was 41.8–53.4 years, with one study not reporting an acetabulum-specific age range. With regards to co-morbidities, none were reported; however, one study described a mean ASA grade of 1.4 (range 1–2) and body mass index (BMI) as an indicator of obesity (mean BMI 23.5 ± 0.4) [32]. The reported mean follow-up time from injury was 33.6 ± 22.4 months for five studies (ranging from 12.0 to 132.0 months).

### 3.4. Initial Acetabulum Injury and Fracture Classification

Two papers described four mechanisms of injury for sustaining an acetabular fracture: RTA, fall (either from standing, low height or high height), work injury, and sporting injury (Table 1) [37,38]. Only one paper presented mean ISS as a marker of concomitant injury [35]. In addition, no papers in this review directly referred to the Judet–Letournel classification of acetabular fractures; however, two papers did present specific fracture patterns [32,37].

### 3.5. Surgical Procedure

All seven studies reported surgical fixation of acetabulum fractures, with one paper additionally noting non-operative management. However, only three studies directly referenced the specific surgical approach; in which the KL approach was used in 44 cases, the IL in 48, the modified Stoppa in 13, and a combined approach in 2 [32,34,35]. The surgical procedures are documented in Table 2 and Table 3.

### 3.6. Outcome Measures

#### 3.6.1. General Outcomes

There were a variety of general outcome measures utilised in this study; these were only reported in the papers relating to sexual function [32,34,37]. Two papers (n = 80) reported quality of fracture reduction using the Matta radiological scoring system [32,34]; of these, 78.8% of patients (63/80) had excellent/good outcomes, with the remaining subjects having fair/poor outcomes. Bony union was reported in one paper, in which 100% of patients had successful union (mean time to union: 3.7 months, range 3–5) [34].

Specific health-related QoL outcomes were measured in two papers. Monteleone et al. (2023) used the Short-Form-12 questionnaire, the Workplace Activity Limitation Scale (WALS), the Harris Hip Score (HHS), and the Tegner score; in this paper, the authors reported that patients had lower functioning scores than the normal population (lower scores in bodily pain and physical role domains), with redundancy (n = 17), and reduced or no sporting involved (n = 18) was noted [37]. In addition, Yavuz et al. (2022) implemented the Merle d’Aubigné Postel (MDP) score to measure overall hip functioning, in which 66.2% (43/65) had excellent scores, 11/65 had good, 7/65 had fair, and 4/65 had poor scores [34]. A Visual Analogue Scale (VAS) was utilised to measure pain in one paper, in which all subjects (15/15) had hip pain at 1 year (mean VAS score 2.9/10) [32].

#### 3.6.2. Sexual Function

A range of outcome measures were reported; all of which were questionnaire-based. Only two studies utilised recognised validated measures for sexual functions outcomes: IIEF-5, FSFI, and IIEF-15 [34,35]. The mean follow-up was 33.5 months (mean range: 12.0 to 65.1 months).

The data suggest that acetabulum fractures have an impact on sexual function ranging from 20.0% to 39.8% within the groups analysed. Frequently described symptoms suggestive of dysfunction included change in sexual activity (n = 33), dyspareunia (n = 1), anorgasmia (n = 1), and reduced erectile function (n = 37). Only one study addressed reasons for decreased sexual function, which included fear of further injury (n = 5), hip pain (n = 4), easy fatiguability (n = 4), decreased libido (n = 3), uncooperative sexual partner (n = 1), and embarrassment from scar (n = 1) [32]. Two papers specifically evaluated sexual satisfaction, which showed reduced scores in both [32,35]. Park et al. (2017) highlighted reasons for reduced sexual satisfaction which mirrored those of reduced sexual function in their study [32]. Only one paper reported on reduced genital function with only 2/21 patients reporting “genital dysfunction” [33].

#### 3.6.3. Urinary Function

Only two papers were found which addressed urinary function [36,38], with a total of 390 patients included. Although the data collection period of these retrospective studies was up to 12 years, specific patient follow-up time was unreported.

Kaneko et al. (2023), in a retrospective study of pelvic and acetabular fracture, reported a low occurrence of spontaneous voiding failure (7/131) [36]. The authors described isolated acetabular fractures as a protective risk factor against spontaneous voiding failure (OR 0.21 [95% CI: 0.09–0.49], *p* < 0.001) and lower urinary tract injuries (OR 0.50 [95% CI: 0.13–1.67], *p* = 0.26); although this latter result did not reach significance. The authors also noted that whilst pelvic fractures were associated with a seven-times increased risk of lower urinary tract injury (*p* = 0.019) and spontaneous voiding failure (*p* < 0.001), there was no increase with acetabular fractures [36].

Jensen et al. (2023) reported on 259 patients with isolated acetabular fractures out of a total cohort of 1061 patients with pelvic fractures [38]. Of these, only one patient (0.4%) sustained a bladder injury. The fracture was sustained from a motor vehicle accident, but it was not recorded what classification type the fracture was, what the surgical approach was and whether the injury was sustained in the accident itself or later surgical management [38].

#### 3.6.4. Complications

There were a variety of post-operative complications noted in the reports included in this study (Table 2 and Table 3). In total, 56 individuals reported complications [33,37,38]. These complications included: neurological deficit (n = 19), post-traumatic osteoarthritis (PTOA) and 12 patients necessitated THA (n = 12), disability on weight-bearing (n = 7), sagging noted on contralateral and ipsilateral hips (n = 6), limp on mobilisation (n = 5), decreased ROM around joint (n = 4), sphincter dysfunction (n = 1), per-pelvic organ dysfunction (n = 1), and urosepsis, urinary retention, sclerosed bladder neck, and permanent suprapubic catheter insertion (n = 1).

## 4. Discussion

Acetabular fractures are an uncommon type of pelvic fracture. Previous research has tended to consider outcomes of these injuries in combination with other pelvic fractures, making it difficult to understand the outcomes of isolated acetabulum fractures independently. This review set out to investigate the sexual and genitourinary functional outcomes in patients following isolated acetabular fractures. The intention was that this would better inform both clinicians and patients about potential fracture- and surgical-related complications as well as identifying any significant gaps in the literature.

The review yielded only seven papers, and all studies were of a cohort design with variable follow-up times [32,33,34,35,36,37,38]. Most involved retrospective reviews of hospital records with subsequent prospective follow-up. A wide range of questionnaires were used to evaluate sexual and genitourinary function; however, only two studies employed recognised validated tools [35,36]. One study focussed entirely on females [33] and one on males [35].

All five sexual function studies demonstrated some change from baseline in terms of sexual function. This included no or decreased sexual activity, dyspareunia, anorgasmia, and change in sexual habit. In men, erectile dysfunction, reduced orgasmic score, and reduced sexual desire was reported [34,35,37]. This is similar to data published on pelvic fractures. A higher frequency of sexual dysfunction was noted in males, although this may be attributed to the fact that more males sustain acetabular fractures [7]. The data did not allow comment on whether more severe dysfunction was associated with biological sex.

With respect to genitourinary dysfunction, there is a severe paucity of available data; however, the two papers reviewed suggest that there is a low risk of impairment [36,38]. Our study described an incidence of “dysfunction” in 8/390 (2.1%) of individuals with acetabular fractures. A limitation of both studies is that there is a lack of acetabular-specific demographic, injury, and procedural data to determine their effects on risk. In addition, there were no validated tools used to report genitourinary outcomes. When compared to pelvic fractures, Kaneko et al. (2023) and an earlier paper by Lefaivre et al. (2022) reported a significantly high risk of urinary dysfunction [36,39]. In the latter paper, urological injury predicted subsequent genitourinary function in the female cohort [39]; however, due to the lack of demographic data provided by Kaneko et al. (2023) and Jensen et al. (2023), this analysis was not possible [36,38].

The strengths of this study lie in the comprehensive search for data, including the use of four electronic search engines. The data were obtained using a structured strategy for data collection and analysis. Data integrity was optimised by discussions of the articles between authors. The located studies included only Level One Major Trauma Centres (MTCs) from a number of countries. All the papers included were published between 2017 and 2023, perhaps representing recent interest in the subject area.

The main limitations of this study relate to the relatively small sample size and incomplete datasets, which have prevented a formal statistical appraisal of the data. The different tools used for data capture, e.g., standardised versus non-standardised patient-reported outcome measures (PROMs) and surgical outcomes, whilst offering complementary information and different perspectives, do not allow direct comparisons of data between studies. It was noted that all urinary function studies utilised objective surgical outcomes and/or complications; in contrast, sexual function was measured using questionnaires. The use of such questionnaires may restrict the answers which patients provide due to the varied and sensitive nature of the subject discussed.

The retrospective capture of baseline data may lead to the potential for missing or limited data and may introduce recall bias. There were also incomplete demographic data reported for each included study, for example, some did not report age or biological sex. None of the studies reported patient co-morbidities, which might have influenced outcomes, and only one reported the ASA grade [32]. Similarly, only one study reported mechanism of injury [37] and one reported ISS [35], respectively, two studies directly reported fracture classification [27,32], and three studies described the surgical approach [32,34,35]. The resulting lack of context prevents a comprehensive understanding of the reasons behind the outcomes of these injuries. The importance of understanding the potential complexities of acetabular and pelvic fractures are underlined by the high rate of medico-legal involvement [40]. Finally, given each of the seven studies represent different patient populations, there will inevitably be a selection bias which will affect the generalisability of the study conclusions.

## 5. Conclusions

In conclusion, this review has highlighted a paucity of data related to outcomes of sexual and genitourinary function in patients who are post-acetabulum fracture. The limited available data suggest that acetabular fractures do have an impact on sexual function, but the impact on genitourinary function is less clear. Further prospective work is required to better understand the relationship between baseline demographics including co-morbidities, injury characteristics such as fracture type, mechanism and concurrent injuries, and surgical fixation and acetabular-fracture outcomes.

## Figures and Tables

**Table 1:** Summary of study, patient, and initial injury characteristics.

Author	Study Characteristics				Patient Characteristics			Initial Acetabulum Injury Characteristics		
	Year of Publication	Country of Publication	Design	No. of Patients	Mean Age (Years) ±SD (Range)	Ratio M:F	Mean ASA Grade ±SD (Range)	Mechanism of Injury	Mean ISS (95% CI)	Fracture Pattern According to Judet-Letournel Classification
Park et al. [32]	2017	South Korea	Prospective cohort	15	42.7 ± 6.9 (30–69)	12:3	1.4 ± 0.5 (1–2)	Unreported	Unreported	6 PW, 2 transverse, 4BC, 2 ACPHT, 1 T-shape
Sadeghpour et al. [33]	2019	Iran	Prospective cohort	21	Mean age unreported (18–49)	0:21	Unreported	Unreported	Unreported	Unreported
Yavuz et al. [34]	2022	Turkey	Prospective cohort	65	41.8 ± 13.0 (18–69)	54:11	Unreported	Unreported	Unreported	Unreported
Elliott et al. [35]	2023	USA	Prospective cohort	92	51.4 ± 14.9	92:0	Unreported	Unreported	15 (12–17)	Unreported
Kaneko et al. [36]	2023	Japan	Retrospective cohort	131	Unreported	Unreported	Unreported	Unreported	Unreported	Unreported
Monteleone et al. [37]	2022	Switzerland	Retrospective cohort	65	53.4 ± 17.6 (20–85)	44:21	Unreported	31 RTA, 30 fall (19 from standing, 3 from low height < 3 m, 8 from high height > 3 m), 2 work injury, 2 ski injury	Unreported	15 AC, 21 PC, 31 BC
Jensen et al. [38]	2023	Denmark	Retrospective cohort	259	Unreported (1 case − age 74)	Unreported (1 case − M)	Unreported	Unreported (1 case − RTA)	Unreported	Unreported (1 case − PW)

SD, standard deviation; M, male; F, female; ASA; American Society of Anaesthesiologists; ISS, Injury Severity Score; CI, confidence interval; PW, posterior wall; BC, both column; ACPHT, anterior column posterior hemi transverse; RTA, road traffic accident; <3 m, less than 3 m; >3 m, greater than 3 m; AC, anterior column; PC; posterior column.

**Table 2:** Characteristics of fracture management and outcome measures, including sexual function.

Author	Management of Acetabulum Fracture			Outcome Measurement			
	Non-Operative Management	Surgical Management	Surgical Approach	Method of Measurement	Tool Used	Matta Radiological Scoring System	Bony Union Achieved (%)
Park et al. [32]	-	Yes	8 KL,5 IL, 2 combined	Questionnaire	VAS pain score, non-validated sexual function questionnaire	8 excellent/good, 7 fair/poor	Unreported
Sadeghpour et al. [33]	Yes (unreported method + individuals)	Yes (unreported method + individuals)	Unreported	Questionnaire, physical examination by orthopaedic surgeon + gynaecologist	Non-validated sexual function questionnaire	Unreported	Unreported
Yavuz et al. [34]	-	Yes	36 KL. 16 IL, 13 modified Stoppa	Questionnaire	IIEF-5, FSFI-5, MDP score	45 excellent, 10 good, 6 fair, 4 poor	100% (mean time to union 3.7 months ± 0.7; range 3.0–5.0)
Elliott et al. [35]	-	Yes	27 IL, 65 unreported	Questionnaire	IIEF-15	Unreported	Unreported
Monteleone et al. [37]	-	65 underwent surgical fixation	Unreported	Questionnaire	Non-validated sexual function questionnaire (scale 1–10 of function), SF-12, WALS, HHS, Tegner score	Unreported	Unreported
**General Outcomes**	**Sexual Outcomes**					**Complications**	**Mean F/U ± SD (Months) (Range)**
**Functional Outcomes**	**Sexual Function**	**Reasons for Change in Sexual Function**	**Sexual Satisfaction**	**Reasons for Sexual Satisfaction**	**Genital Function**		
15 hip-pain at 1 year, mean VAS score 2.9/10 (range 1–7)	14/15 resumed sexual activity within 1-year (mean time 3.9 months; range 2–6); 11/14 had decreased frequency of sexual activity	5 with fear of further injury, 4 hip pain, 4 easy fatiguability, 3 decreased libido, 1 uncooperative sexual partner, 1 embarrassment from scar	7 had decreased/greatly decreased sexual satisfaction	5 easy fatiguability, 4 fear of further injury, 3 decreased libido, 2 hip pain, 1 embarrassment from scar	Unreported	Unreported	12.0
Unreported	16/21 normal, 2/21 reduced frequency of sexual contact, 1/21 dyspareunia, 1/21 anorgasmia, 1 unreported, 0 coital incontinence, 0 painful orgasm	Unreported	Unreported	Unreported	17 normal, 1 dysmenorrhoea, 1 pelvic pain, 0 pelvic organ prolapse, 0 vaginal prolapse feeling	7 had disability on WB, 6 had sagging contralateral + ipsilateral hip, 4 had decreased ROM, 5 had limp	12.0
MDP score: 43 excellent, 11 good, 7 fair, 4 poor	IIEF-5: significantly reduced scores (24.3 ± 1.8 to 20.0 ± 4.8) FSFI-5: significantly reduced scores (24.9 ± 6.3 to 18.3 ± 6.2)	Unreported	Unreported	Unreported	Unreported	No complications	36.3 ± 7.8 (24.0–54.0)
Unreported	37/92 developed moderate- severe erectile dysfunction, reduced complete IEF score (59.5 to 49.6), reduced EF score (24.8 to 19.3), reduced orgasmic score (8.7 to 7.8), reduced sexual desire score (8.2 to 7.4)	Unreported	Significantly reduced intercourse satisfaction score (10.4 to 8.7), reduced overall satisfaction score (7.9 to 6.4)	Unreported	Unreported	Unreported	42.3 (minimum 12.5)
Lower functioning than normal population (worse scores in bodily pain + physical role), 17 became redundant, 18 had reduced/no sport involvement	16/44 males + 4/21 females reported change in sexual habit	Unreported	Unreported	Unreported	Unreported	19 with neurological deficit, 12 had PTOA (underwent THA), 1 sphincter dysfunction, 1 per-pelvic organ dysfunction	65.1 ± 36.4 (12.0–132.0)

%, percentage; F/U, follow-up; SD, standard deviation; KL, Kocher–Langenbeck approach; IL, ilioinguinal approach; VAS, Visual Analogue Scale; WB, weight-bearing; ROM, range of movement; SF-12, Short-Form-12; WALS, Workplace Activity Limitation Scale; HHS, Harris Hip Score; PTOA, post-traumatic osteoarthritis; THA, Total Hip Arthroplasty; IIEF-5, Internation Index of Erectile Function-5; FSFI-5, Female Sexual Function Index-5; MDP score, Merle d’Aubigné Postel score; IIEF-15; EF, International Index of Erectile Function-15, erectile function.

**Table 3:** Characteristics of fracture management and outcome measures, including genitourinary function.

Author	Management of Acetabulum Fracture			Outcome Measurement		General Outcomes			Genitourinary Outcomes		Complications	Mean F/U ± SD (Months) (Range)
	Non-Operative Management	Surgical Management	Surgical Approach	Method of Measurement	Tool Used	Matta Radiological Classification	Bony Union Achieved (%)	Functional Outcomes	Urinary Function	Urinary Satisfaction		
Kaneko et al. [36]	-	Yes	Unreported	Surgical outcomes	-	Unreported	Unreported	Unreported	7/131 without spontaneous voiding, fracture was protective against spontaneous voiding failure (*p* < 0.001), fracture was protective against lower urinary tract injuries (*p* = 0.26)	Unreported	Unreported	Unreported
Jensen et al. [38]	-	Yes	Unreported	Surgical outcomes	-	Unreported	Unreported	Unreported	1/259 bladder injury	Unreported	1/259 urosepsis, urinary retention, sclerosed bladder neck + permanent SPC inserted	Unreported

F/U, follow-up; SD, standard deviation; SPC, suprapubic catheter.

## Data Availability

The data presented in this study are available on reasonable request from the corresponding author (PVG).

## Supplementary Materials

The following supporting information can be downloaded at https://www.mdpi.com/article/10.3390/jcm14010230/s1, Table S1: Table describing the completed PRISMA 2020 Checklist [41]; Table S2: Table showing the Medline, CENTRAL, Scopus, and EMBASE database search protocols used; Table S3: OCEBM and GRADE Methodological Systems.
