# Supplementary material for: Sexual and Urinary Dysfunction Following Isolated Acetabulum Fractures: A Systematic Review of the Literature
Source: J Clin Med. 2025 Jan 3;14(1):230. doi: 10.3390/jcm14010230 (PMC11721351; doi:10.3390/jcm14010230)
Supplement: Supplementary file 1 [file jcm-14-00230-s001.zip › Table S2.pdf]

Table S2. Table showing the Medline, CENTRAL, Scopus and EMBASE database search protocols used.

MEDLINE	
**Search**	**Number of Results**
1.) "acetabulum" OR "acetabular"	25984
2.) "fracture"	386809
3.) "sexual function*" OR "erectile function*" OR "urinary function*"	220365
4.) 1, 2 AND 3	22
**CENTRAL**	
**Search**	**Number of Results**
1.) "acetabulum" OR "acetabular"	1171
2.) "fracture"	30443
3.) "sexual function*" OR "erectile function*" OR "urinary function*"	19986
4.) 1, 2 AND 3	2
**SCOPUS**	
**Search**	**Number of Results**
1.) "acetabulum" OR "acetabular"	73266
2.) "fracture"	2102701
3.) "sexual function*" OR "erectile function*" OR "urinary function*"	694568
4.) 1, 2 AND 3	428
**EMBASE**	
**Search**	**Number of results**
1.) "acetabulum" OR "acetabular"	32712
2.) "fracture"	412528
3.) "sexual function*" OR "erectile function*" OR "urinary function*"	228587
4.) 1, 2 AND 3	62
