# Supplementary material for: Sexual and Urinary Dysfunction Following Isolated Acetabulum Fractures: A Systematic Review of the Literature
Source: J Clin Med. 2025 Jan 3;14(1):230. doi: 10.3390/jcm14010230 (PMC11721351; doi:10.3390/jcm14010230)
Supplement: Supplementary file 1 [file jcm-14-00230-s001.zip › Table S3.pdf]

Table S3. OCEBM and GRADE Methodological Systems.

All primary research articles obtained for this systematic review were prospective cohort [32-25] and retrospective cohort [36-38] studies. There are currently no large cohort studies or randomised controlled trials (RCTs) describing sexual and urinary function following isolated acetabular fractures.

**Author**	**Design of study**	**OCEBM Level of Evidence** [30]	**GRADE Assessment** [31]
Park et al. [32]	Prospective cohort	Level II	Low
Sadeghpour et al. [33]	Prospective cohort	Level II	Low
Yavuz et al. [34]	Prospective cohort	Level II	Low
Elliott et al. [35]	Prospective cohort	Level II	Low
Kaneko et al. [36]	Retrospective cohort	Level III	Low
Monteleone et al. [37]	Retrospective cohort	Level III	Low
Jensen et al. [38]	Retrospective cohort	Level III	Low
